# ‘nAb’ the self-reactive activity in the COVID-19 combat

**DOI:** 10.1038/s41392-021-00518-2

**Published:** 2021-03-02

**Authors:** Madhusudhanan Narasimhan, Lenin Mahimainathan, Alagarraju Muthukumar

**Affiliations:** Department of Pathology, University of Texas Southwestern Medical Center, Dallas, TX USA

**Keywords:** Infectious diseases, Experimental models of disease

**Fig. 1:**
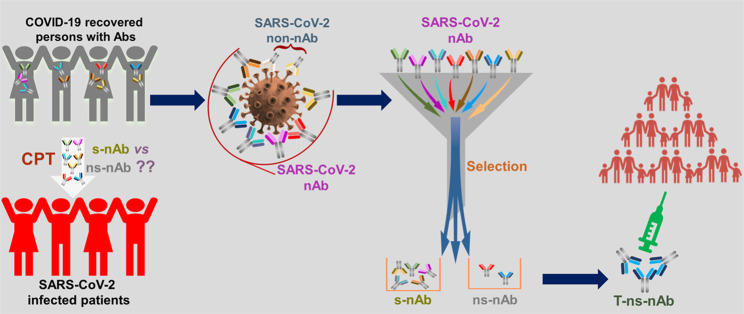
Non-self-reacting antibodies and SARS-CoV-2 infection. Do the passive antibody supply strategy-based CPT require exclusion of autoreactive and non-nAbs, and retention of Abs with non-self-reactive-neutralizing activity to improve efficacy? (left). Non-self-reactive-neutralizing antibody proposed to be a potent therapy for inhibiting the SARS-CoV-2 activity (right). CPT convalescent plasma therapy, nAb neutralizing antibody, s-nAb self-reactive-neutralizing antibody, ns-nAb non-self-reactive-neutralizing antibody, T-ns-nAb therapeutic non-self-reactive antibody

The recent article in Cell by Kreye et al.^1^ discovered that non-self-reactive-neutralizing antibody (ns-nAb) could be an effective way to combat SARS-CoV-2 infection. Their observations provide new perspectives for therapeutic antibody engineering to consider non-self-reactivity as part of the process to improve its selection and potency (Fig. 1).

As we deal with the prevailing burden of COVID-19 crisis, the current viral resurgence is threatening to drive many walks of life to the brinks and further the insult. This emphasizes the immediate need for therapeutic and prophylactic treatment modalities to combat the pandemic. Several treatment options such as vaccines, monoclonal antibodies, anti-virals, and convalescent plasma therapy (CPT) are under evaluation and a few frontrunners have shown promising immune response. Central to many of these treatment repertoires, are neutralizing antibodies (nAbs) that play an important role in the body’s immune response to render the virus incompetent to attach and/or penetrate the host cells. Therefore, a comprehensive understanding of the nAbs becomes urgent. Thus far, LY-CoV555, JS016, REGN-COV2, TY027, BRII-196, BRII-198, CT-P59, and SCTA01 are some of the nAbs against SARS-CoV-2 to enter clinical testing.

Now, in their study in *Cell*, Kreye et al.^1^ has shed a new light that engineered nAb can hold the promise to offer prompt pandemic response when we are amidst the early phase distribution of effective vaccines from Pfizer, Moderna, and Sinovac. Elegant structural and functional studies have been used to screen the blood of COVID-19 survivors for potential neutralizing SARS-CoV-2 antibodies. Out of 598 antibodies screened, 18 nAbs were identified to be effective in neutralizing the SARS-CoV-2 from entering cells and replicating. Importantly, a few of the near-germline SARS-CoV-2 nAbs reacted with mammalian self-antigens. Upon further cross-reactivity analysis, only CV07-209 showed the strongest neutralizing and non-self-reactive (ns) neutralizing activity. Further, the immunochemistry and histological data buttressed that the prophylactic and therapeutic efficacy of CV07-209 can alleviate the SARS-CoV-2-induced clinical signs of lung lesions besides leading to maintenance of healthy weight in the pre-clinical hamster model. Overall, Kreye et al.^1^ is foremost to emphasize the need to evaluate the cross-reactivity of nAb for self- and non-self-reactive attribute in SARS-CoV-2 infection therapies.

The non-self-reactive knowledge described in the SARS-CoV-2 context here is worth mentioning, especially during this early excitement of monoclonal therapies and COVID-19 vaccine’s rollout. Some SARS-CoV-2 nAbs engenders self-reactive antibodies (s-nAbs) that can react with body’s own antigens and attack its own cells or tissues leading to auto-immune-related detrimental effects.^2–4^ Since vaccine (antigen)-mediated elicitation of antibodies and monoclonal antibody therapies are expected to neutralize the SARS-CoV-2, any unintended self-reactions could annul the benefit and spiral into lasting health crisis. In addition, the self-reactive activity can exert an immense influence in CPT supportive care, a passive antibody transference strategy that leverages immune factors found in the plasma derived from the blood of donors recovered from illness (here, COVID-19). Not known is the readiness of reliable testing strategies confirming the convalescent plasma (CP) for the exclusion of undesired s-Abs and retention of only the key ns-nAbs. Thus, in the hindsight, this study can be a guide to therapeutic nAb (T-nAb) engineers to screen specially for the ns-nAbs in their process of generating efficient immunotherapeutic molecules. Such screening/selection strategies supported by detailed validation studies could form a basis for the policy makers to reflect on their mandates regarding self-reactive/non-self-reactive-neutralizing activity in qualifying CP or T-nAbs landscape in the COVID-19 combat. Notably, this compelling non-self-reactive aspect can exert far-reaching influence in other infectious diseases, as well.

Of relevance, nAbs will continue to play a vital complementary role post-availability of vaccine(s) to accommodate a subgroup that is vaccine non- and low- responders, anti-vaxxers, or unvaccinated owing to age, co-morbid conditions, or fear of adverse effects, who may remain to spread the disease actively, if not attended. Also, nAbs could play a significant role in developing world that may encounter vaccine wait because of affordability and accessibility quandaries. Given this and COVID-19 is being speculated to be an endemic or a circulating disease, akin to annual influenza,^5^ sensible therapy/management options are indispensable to either complement or take over the lead, contextually. Also, since nosocomial transmission can endanger the supporting caregivers and frontline healthcare workers with inevitable high-risk exposure to a diagnosed COVID-19 patient, a putative therapeutic application of ns-nAb treatment of COVID-19 patients and prophylactic treatment of caregivers could be worth considering. Apparently, as with any evolving concepts, it warrants a continued research refinement to assess its fullest merits in clinical use.

While nAbs are being on the therapy radar to bring hope to thwart the viral invaders, this study has kindled interest to explore the following aspects:

1. Are non-self-reactive T-nAbs the suitable choice for COVID-19 mitigation? If so, what testing measures are essential and/or available to exclude self-reactive nAbs and improve the immunological characterization of the T-nAbs?
2. How to design a unified strategy in the selection, testing, and dosing benchmark(s) of T-nAbs applicability to the broad population when there is a likelihood of inter-individual variability of T-nAbs potency?
3. How does the T-nAb alter the patients’ overall immune response and how durable is the neutralizing activity? This is important to comprehend their clinical efficacy and ascertain whether the T-nAb-treated recovered patients will be immune against SARS-CoV-2 reinfection?
4. What are the measures to stem the risk of antibody-dependent enhancement (ADE) that can facilitate the virus infectivity and survival leading to more severe infections?
5. Is it indispensable to follow up or monitor for self-reactive antibodies and post-trial autoimmunity clinical phenotypes in the COVID-19 antibody clinical trials?

Underlying these interests, it is imperative to consider the aspects linked to immunogenicity and response, viz., the dose, frequency, route, and time of administration (how early or severe the patients are into the infection/disease course?).

In summary, the results presented by Kreye et al.^1^ revealed for the first time that a prophylactic and therapeutic application of CV07-209, a COVID-19 patient-derived ns-nAb conferred protection against SARS-CoV-2-induced bronchopulmonary pathology in a hamster model. Also, given that the nAbs are receiving intense attention, fast tracking of funding and trials, and expedited emergency use authorizations (EUA), this report is germane for the COVID-19 scientific community to consider applying rigorous nAb standards with respect to non-self-reactivity prior to its deployment. After all, a well-founded research practice and technical improvement is pivotal to circumvent the efficacy and safety-related bottlenecks and optimize the benefit of a therapy.
